# Supplementary material for: A highly efficient human cell-free translation system
Source: bioRxiv. 2023 May 23:2023.02.09.527910. Originally published 2023 Feb 10. Preprint. [Version 2] doi: 10.1101/2023.02.09.527910 (PMC9934684; doi:10.1101/2023.02.09.527910)
Supplement: Supplement 1 [file NIHPP2023.02.09.527910v2-supplement-1.pdf]

bioRxiv preprint doi: https://doi.org/10.1101/2023.02.09.527910; this version posted May 23, 2023. The copyright holder for this preprint (which was not certified by peer review) is the author/funder, who has granted bioRxiv a license to display the preprint in perpetuity. It is made available under aCC-BY 4.0 International license.

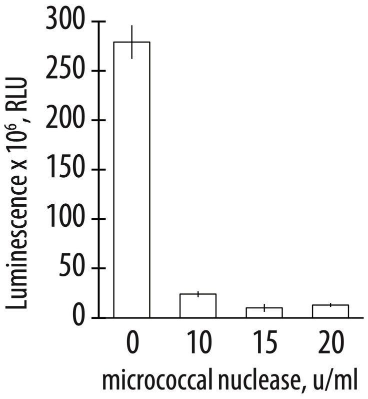

Figure S1. Translational activity of the human extract pretreated with micrococcal nuclease. Extracts were treated with the indicated concentrations of micrococcal nuclease prior to carrying out translation reactions with nanoluciferase mRNA. All error bars represent one standard deviation of three independent replicates.
